# How did the mental health symptoms of children and adolescents change over early lockdown during the COVID‐19 pandemic in the UK?

**DOI:** 10.1111/jcv2.12009

**Published:** 2021-04-28

**Authors:** Polly Waite, Samantha Pearcey, Adrienne Shum, Jasmine A. L. Raw, Praveetha Patalay, Cathy Creswell

**Affiliations:** ^1^ Department of Experimental Psychology University of Oxford Oxford UK; ^2^ Department of Psychiatry University of Oxford Oxford UK; ^3^ School of Psychology and Clinical Language Sciences University of Reading Reading UK; ^4^ Faculty of Population Health Sciences Centre for Longitudinal Studies and MRC Unit for Lifelong Health and Ageing University College London London UK

**Keywords:** adolescent, children, COVID‐19, lockdown, mental health, pandemic, United Kingdom, youth

## Abstract

**Background:**

The COVID‐19 pandemic has caused extensive disruption to the lives of children and young people. Understanding the psychological effects on children and young people, in the context of known risk factors is crucial to mitigate the effects of the pandemic. This study set out to explore how mental health symptoms in children and adolescents changed over a month of full lockdown in the United Kingdom in response to the pandemic.

**Methods:**

UK‐based parents and carers (*n* = 2673) of school‐aged children and young people aged between 4 and 16 years completed an online survey about their child's mental health at two time points between March and May 2020, during early lockdown. The survey examined changes in emotional symptoms, conduct problems and hyperactivity/inattention.

**Results:**

The findings highlighted particular deteriorations in mental health symptoms among preadolescent children, which translated to a 10% increase in those meeting possible/probable caseness criteria for emotional symptoms, a 20% increase in hyperactivity/inattention, and a 35% increase in conduct problems. In contrast, changes among adolescents were smaller (4% and 8% increase for hyperactivity/inattention and conduct problems, respectively) with a small reduction in emotional symptoms (reflecting a 3% reduction in caseness). Overall, there were few differences in change in symptoms or caseness over time according to demographic characteristics, but children and young people in low income households and those with special educational needs and/or neurodevelopmental disorders exhibited elevated symptoms (and caseness) at both time points.

**Conclusions:**

The findings highlight important areas of concern in terms of the potential impact of the first national lockdown on children and young people's adjustment. Developing an understanding of who has been most severely affected by the pandemic, and in what ways, is crucial in order to target effective support where it is most needed.

## Graphical abstract

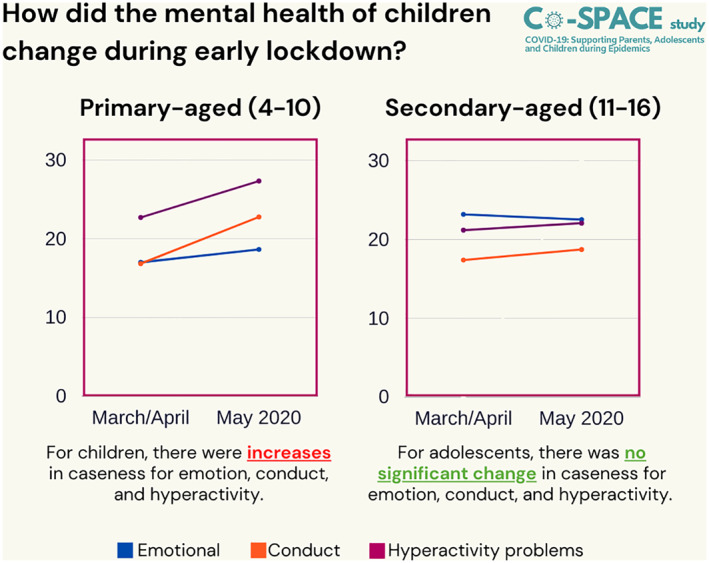

## INTRODUCTION

While children and young people are at low risk of infection from coronavirus disease 2019 (COVID‐19), the pandemic and the measures taken to try to minimise the spread of the virus, such as lockdown, school closures and social distancing, have caused extensive disruption to the lives of children and young people. Understanding the psychological effects of the COVID‐19 pandemic on children and young people, in the context of known risk factors is crucial to mitigate the effects of the pandemic (Holmes et al., 2020).

**Key points**

- Early cross‐sectional findings have given an indication that children and young people have had relatively high levels of mental health symptoms during the COVID‐19 pandemic.
- Little is known about changes in children and young people's mental health in the United Kingdom during the first national lockdown.
- The findings highlighted particular deteriorations in mental health symptoms over a month during early lockdown among preadolescent children.
- There were elevated symptoms at both time points (but little change over time) for children from low income households and those with special educational needs and/or neurodevelopmental disorders.
- Developing an understanding of who has been most severely affected by the pandemic, and in what ways, is crucial in order to target effective support where it is most needed.

Early cross‐sectional findings have given an indication that children and young people have had relatively high levels of mental health symptoms during the pandemic (Racine et al., 2020). For example, in China, Xie et al. (2020) found that 22.6% of 2330 young people survey reported elevated depressive symptoms and 18.9% reported elevated anxiety symptoms during lockdown. We have also recently started to see reports based on comparisons between children and young people's mental health prior to the pandemic and at a particular point of time during the pandemic. Of particular note, the NHS Digital Survey of children and young people's mental health in England (NHS Digital, 2020) highlighted that in July 2020 (after the end of national lockdown but while many restrictions were still in place) the proportion of children and young people with a probable mental health disorder was one in six, compared to one in nine in 2017. While it is possible that this deterioration may have been a continuation of the pattern that had been seen from previous surveys (Sadler et al., 2018), the fact that over 40% of young people reported that they felt that the pandemic had made their mental health worse highlights the potential contribution of the pandemic to this worsening picture. However, the lack of longitudinal data on change over time during the pandemic limits our understanding of how particular features of the pandemic, such as national lockdown (which included school closures for most children), were associated with changes in mental health.

It is likely that the impact of the pandemic will differ depending on a range of factors, including those already known to be risk factors for poor mental health generally. For children and young people, this includes being from a low income household (Gutman et al., 2015; Wickham et al., 2017), a single parent household (partly due to material disadvantage) (Dunn et al., 1998; Spencer, 2005), and having special educational needs (SEN) that require special health and education support (Gadeyne et al., 2004; Linna et al., 1999). Indeed, there are already indications of a high prevalence of emotional and behavioural difficulties among young people with neurodevelopmental disorders (NDs) during early lockdown (Nonweiler et al., 2020). In general, there are also differences in the risk of developing mental health difficulties on the basis of age and gender, with boys of primary school age more likely to have any mental disorder (12.2%; most commonly behavioural problems) than girls of the same age (6.6%), but by secondary school age, boys and girls are equally likely to have any mental disorder with higher rates of emotional disorders among adolescent girls (Davis et al., 2018). Finally, the impact of the pandemic may have differed between age groups. For example, compared to adolescents, younger children may have faced particular disruption given that they are likely to be less able to access learning independently while out of school, are more dependent on their parents (who are known to have experienced high levels of stress during lockdown; (Office for National Statistics, 2020), and less able to connect with peers in meaningful ways (e.g., remotely through electronic devices rather than face to face play). However, adolescents might be particularly affected due to their normative drive for autonomy and social connections (Steinberg, 1990), which were curtailed during lockdown.

The Co‐SPACE (COVID‐19: supporting parents, adolescents and children during epidemics) study was set up to track the trajectories of mental health of children and young people during the COVID‐19 pandemic in the United Kingdom through a monthly online survey completed by parents and carers of children and young people aged 4–16 years. In this paper, we set out to answer the following research questions:

1. How did mental health of participating children and adolescents change during early lockdown in the UK—in terms of both continuous symptoms and ‘caseness’?
2. How did this vary on the basis of (i) child gender, (ii) household income (living in poverty or not) and family composition (i.e., single adult family or not), and (iii) presence of SENs/NDs?

This early lockdown period in the United Kingdom involved a national lockdown from the end of March 2020 (including across the devolved nations), during which schools were closed (except to children of key workers and vulnerable children), people were not allowed to mix with others outside their household, nonessential shops, entertainment venues and playgrounds were closed, and people were instructed to stay at home except for very limited purposes (e.g., food shopping). Restrictions began to be eased across the UK from the beginning of June 2020.

## METHODS

### Participants

Parents and carers (over the age of 18 years) of school‐aged children and young people aged between 4 and 16 years who lived in the United Kingdom were eligible to take part. The current paper focuses on the 2673 participants who completed the baseline survey online between the 30th March and the 30th April 2020 and a follow‐up survey 1 month after baseline (30th April 2020–30th May 2020), and completed the Strengths and Difficulties Questionnaire (SDQ; Goodman, 1997; Goodman et al., 1998) at both time points. Demographic information for participants and their children can be found in Table 1.

**TABLE 1:** Sample demographics

	Children (4–10 years)	Adolescent (11–16 years)	Full sample
	*n* = 1776 (66.44%)	*n* = 897 (33.56%)	*n* = 2673
Parent gender			
Male	107 (6.03%)	43 (4.79%)	150 (5.61%)
Female	1664 (93.70%)	849 (94.65%)	2513 (94.01%)
Parent ethnicity			
White British	1698 (95.61%)	871 (97.10%)	2569 (96.11%)
Other	78 (4.39%)	26 (2.90%)	104 (3.89%)
Parent/carer education			
School/vocational qualification	247 (13.91%)	145 (16.17%)	392 (14.67%)
Undergraduate degree	702 (39.53%)	366 (40.80%)	1068 (39.96%)
Postgraduate degree	819 (46.12%)	376 (41.92%)	1195 (44.71%)
Child mean age (SD)	7.10 (1.90)	13.31 (1.67)	
Child gender			
Male	916 (51.58%)	466 (51.95%)	1382 (51.70%)
Female	853 (48.03%)	418 (46.60%)	1271 (47.55%)
Child ethnicity			
White British	1631 (91.84%)	848 (94.54%)	2479 (92.74%)
Other	145 (8.16%)	49 (5.46%)	194 (7.26%)
Child SEN/ND	243 (13.68%)	208 (23.19%)	451 (16.87%)
Household income			
<£16,000 p.a.	79 (4.45%)	61 (6.80%)	140 (5.24%)
>£16,000 p.a.	1588 (89.41%)	755 (84.17%)	2343 (87.65%)
Prefer not to say	109 (6.14%)	81 (9.03%)	190 (7.11%)
Family composition			
Single adult household	206 (11.60%)	155 (17.28%)	361 (13.51)
Multiple adult household	1570 (88.40%)	742 (82.72%)	2312 (86.50)

Abbreviation: SEN/ND, special educational needs/neurodevelopmental disorders.

### Recruitment

Participants were recruited through a variety of means, including promoting the study through partner organisations, networks, charities and schools, print and digital media coverage and social media.

### Procedure

Parents/carers provided informed consent and then completed the baseline survey online between 30th March and the 29th April 2020 and a follow‐up survey 1 month after baseline (30th April 2020–30th May 2020). If participants had more than one child within this age range, they were asked to choose one ‘index’ child to report on each time. A link to the follow up survey was sent via email to each parent/carer one calendar month after they had completed their baseline survey. Full procedural information can be found in the protocol (osf.io/8zx2y). Ethical approval for the study was provided by the University of Oxford Medical Sciences Division Ethics Committee (reference R69060).

### Measures

*Demographics*. Parents/carers reported on their own and their child's age, gender, and ethnicity and on their total household income. Due to the typical differences in patterns of child and adolescent mental health and their different educational experiences, we dichotomised age at baseline as 4–10 year olds (children) and 11–16 year olds (adolescents). A household income of less than £16,000 per year was categorised as ‘low household income’ as it reflects an income below 60% of the median income in the United Kingdom. Parents/carers were asked whether or not their child had a SEN and/or diagnosed attention deficit hyperactivity disorder (ADHD) or autism spectrum disorder (ASD). Parents/carers were also asked about their family composition (to establish whether there were any other adults aged 18 years or older living in the household).

*SDQ* (Goodman, 1997; Goodman et al., 1998). The mental health of children and young people in the survey was measuring using the SDQ, a brief behavioural screening questionnaire. This measure has been validated in both community and clinical samples and is able to detect psychiatric diagnoses with good sensitivity and specificity (Goodman et al., 2000; Stone et al., 2010). The parent/carer‐report version was used due to its satisfactory psychometric properties across the study age range (Stone et al., 2010). The SDQ consists of 25 items, each rated on a 3‐point Likert scale ranging from 0 (‘not at all’) to 2 (‘certainly true’). There are five subscales, each consisting of five items, assessing emotional symptoms, conduct problems, hyperactivity/inattention, peer relationship problems and prosocial behaviour. In the current paper, we examine the three subscales that relate to mental health symptoms: emotional symptoms (related to fear/worry, clinginess, sadness and somatic symptoms), conduct problems and hyperactivity/inattention. A subscale score is obtained by summing the responses in each of the subscales (range: 0–10). Where there was missing data, the person mean was imputed on responses to at least three of the five subscale items. The SDQ also includes an impact supplement which assesses the functional impairment of the identified problems across four domains (the child's home life, friendships, school‐life and leisure activities) and distress. Impact items are scored on a four point scale from 0 if either ‘not at all’ or ‘only a little’, 1 if ‘quite a lot’ and 2 if ‘a great deal’. Scores on the impairment and distress items are totalled, leading to a maximum total impact score of 10. As is a standard requirement for the SDQ, at the first assessment the SDQ asked about symptoms and impact over the last 6 months, and follow‐up assessments asked about the preceding month.

The likelihood that a child or young person may have a mental disorder can be classified using a pseudo diagnostic algorithm as ‘unlikely’, ‘possible’ or ‘probable’, based on both symptom (>80th percentile = possible) and impact (‘quite a lot’ in at least one domain = possible) ratings (Goodman, 1999; Goodman et al., 2000). In this study we followed the ‘lenient’ approach used by Nielsen et al. (2019) with preadolescent children, distinguishing between ‘possible’/‘probable’ and ‘unlikely’ cases, to err on the side of being inclusive to those who might be a potential ‘case’.

### Data analysis

All analyses were carried out in R Studio (v. 1.3.1093) using R (version 4.0.3). We calculated SDQ caseness categories using syntax downloaded from: http://www.sdqinfo.com/py/sdqinfo/c0.py. To examine change over time, the main effect of time point on SDQ symptoms was examined within separate linear mixed effects models for children and adolescents, and on SDQ caseness within binomial generalised mixed effects models (using a bobyqa optimizer, unless stated otherwise). We next repeated the models above, first with the inclusion of each variable of interest individually (where they were not already included in the models as covariates) and again with those variables as an interaction with time point, to establish how patterns of change in mental health symptoms varied on the basis of (i) child gender, (ii) household income (low [poverty level] income or not) and family composition (i.e., single adult family or not), (iii) presence of SENs/NDs (including ASD and ADHD). Models were run using the lmer function within the lme4 package (v. 1.1‐23; Bates et al., 2015). Each model was estimated using maximum likelihood estimations (with laplace approximation for caseness models) and included dichotomous variables of child age, gender, and ethnicity and total household income and employment status as fixed effects. A random intercept was included for each participant and time was treated as a dichotomous variable with ‘0’ representing baseline (baseline = 0; follow‐up = 1).

## RESULTS

Question 1. How did mental health of participating children and adolescents change during early lockdown in the United Kingdom—in terms of both continuous symptoms and ‘caseness’?

Table 2 presents the model results of the main effects of time for parent/carer reported emotional symptoms, conduct problems, and hyperactivity/inattention and caseness. All means, percentages and confidence intervals can be found in Tables S1 and S2 (available as an online data supplement).

**TABLE 2:** Model results from separate regression models estimating the main effects of time and other key predictors of parent/carer reported emotional, conduct and hyperactivity/inattention symptoms and problems, with significance values for their interaction with time point

	SDQ emotion				SDQ conduct				SDQ hyperactivity/inattention			
SDQ symptom models												
	*b* (SE)/	*t*‐Value/	95% CI	Time* predictor interaction	*b* (SE)	*t*‐Value	95% CI	Time*predictor interaction	*b* (SE)	*t*‐Value	95% CI	Time* predictor interaction
Children (aged 4–10 years)												
Time point^1^	0.14 (0.04)	3.23**	[0.05, 0.22]	–	0.30 (0.03)	9.62***	[0.24, 0.36]	–	0.61 (0.04)	13.86***	[0.52, 0.69]	–
Gender^1^	−0.24 (0.11)	−2.18*	[−0.46, −0.02]	*p* = 0.79	0.24 (0.08)	3.01**	[0.08, 0.40]	*p* = 0.03	0.97 (0.12)	8.13***	[0.73, 1.20]	*p* = 0.09
Income^1^	1.33 (0.27)	4.88***	[0.79, 1.86]	*p* = 0.68	0.87 (0.20)	4.43***	[0.48, 1.25]	*p* = 0.76	1.17 (0.29)	3.99***	[0.59, 1.74]	*p* = 0.76
Single Adult^2^	0.69 (0.18)	3.74***	[0.33, 1.04]	*p* = 0.93	0.19 (0.13)	1.41	[−0.07, 0.45]	*p* = 0.28	0.52 (0.20)	2.61**	[0.13, 0.90]	*p* = 0.14
SEN/ND^3^	2.21 (0.16)	14.04***	[1.90, 2.52]	*p* = 0.01	1.67 (0.11)	14.85***	[1.45, 1.89]	*p* < 0.001	2.97 (0.16)	18.14***	[2.65, 3.29]	*p* < 0.001
Adolescents (aged 11–16 years)												
Time point^4^	−0.24 (0.06)	−4.04***	[−0.36, −0.13]	–	0.02 (0.04)	0.63	[−0.05, 0.10]	–	0.12 (0.06)	2.15*	[0.01, 0.24]	–
Gender^4^	−0.76 (0.17)	−4.50***	[−1.10, −0.43]	*p* = 0.56	0.28 (0.12)	2.30*	[0.04, 0.51]	*p* = 0.04	1.15 (0.17)	6.75***	[0.82, 1.48]	*p* = 0.49
Income^4^	1.19 (0.35)	3.42***	[0.51, 1.87]	*p* = 0.16	0.38 (0.25)	1.53	[−0.11, 0.86]	*p* = 0.69	0.97 (0.35)	2.79**	[0.29, 1.65]	*p* = 0.80
Single Adult^5^	0.06 (0.25)	0.24	[−0.42, 0.54]	*p* = 0.02	0.11 (0.17)	0.65	[−0.23, 0.45]	*p* = 0.48	0.17 (0.25)	0.68	[−0.31, 0.65]	*p* = 0.51
SEN/ND^6^	2.41 (0.19)	12.50***	[2.03, 2.79]	*p* = 0.48	1.40 (0.14)	10.06***	[1.13, 1.68]	*p* = 0.004	2.65 (0.19)	13.96***	[2.28, 3.02]	*p* = 0.001
SDQ problems models												
	OR (SE)	*z*‐Value	95% CI	Time* predictor interaction	OR (SE)	*z*‐Value	95% CI	Time* predictor interaction	OR (SE)	*z*‐Value	95% CI	Time* predictor interaction
Children												
Time point^7^	1.68 (0.32)	2.71**	[1.15, 2.46]	–	5.10 (1.05)	7.95***	[3.41, 7.63]	–	1.34 (0.20)	6.61***	[0.94, 1.74]	–
Gender^7^	0.97 (0.33)	−0.08	[0.50, 1.91]	*p* < 0.001	1.49 (0.47)	1.27	[0.81, 2.76]	*p* = 0.01	0.81 (0.35)	2.34*	[0.13, 1.49]	*p* < 0.001
Income^7^	8.08 (7.10)	2.38*	[1.44, 45.25]	*p* = 0.92	4.71 (3.84)	1.90	[0.95, 23.24]	*p* = 0.53	2.34 (1.19)	1.97	[0.01, 4.68]	*p* = 0.23
Single Adult^8^	1.63 (0.88)	0.90	[0.56, 4.71]	*p* = 0.45	1.49 (0.75)	0.80	[0.56, 4.00]	*p* < 0.001	1.35 (0.78)	0.52	[0.44, 4.18]	*p* = 0.27
SEN/ND^9,^ ^a^	–	8.01***	–	*p* < 0.001	–	7.87***	–	*p* < 0.001	–	22.60***	–	*p* < 0.001
Adolescents												
Time point^10^	0.76 (0.20)	−1.05	[0.45, 1.27]	–	0.53 (0.29)	1.82	[−0.04, 1.09]	–	1.40 (0.41)	1.16	[0.79, 2.49]	–
Gender^10^	0.54 (0.27)	−1.24	[0.20, 1.44]	*p* = 0.51	0.45 (0.52)	0.87	[−0.57, 1.47]	*p* = 0.12	2.15 (1.20)	1.38	[0.72, 6.40]	*p* = 0.99
Income^10^	7.14 (8.07)	1.74	[0.78, 65.36]	*p* = 0.06	0.09 (0.99)	0.09	[−1.85, 2.02]	*p* = 0.55	2.80 (2.81)	1.03	[0.39, 19.97]	*p* = 0.19
Single Adult^11^	1.00 (0.73)	−0.01	[0.24, 4.22]	*p* = 0.68	0.92 (0.69)	−0.12	[0.21, 3.99]	*p* = 0.24	1.20 (0.93)	0.24	[0.26, 5.47]	*p* = 0.05
SEN/ND^12,^ ^a^	–	5.6***	–	*p* = 0.16	–	3.58***	–	*p* < 0.001	–	14.41***	–	*p* < 0.001

*Note:* Data for main effects taken from the same models are denoted with the same number in superscript. All interaction *p*‐values are from individual and separate models.

Abbreviations: CI, confidence interval; SDQ, Strengths and Difficulties Questionnaire; SEN/ND, special educational needs/neurodevelopmental disorders.

^a^ Meaningful odds ratios for the main effect of SEN/ND could not be calculated due to the significant overlap between caseness and SEN/ND.

*p < 0.05, **p < 0.01, ***p < 0.001.

**FIGURE 1:**
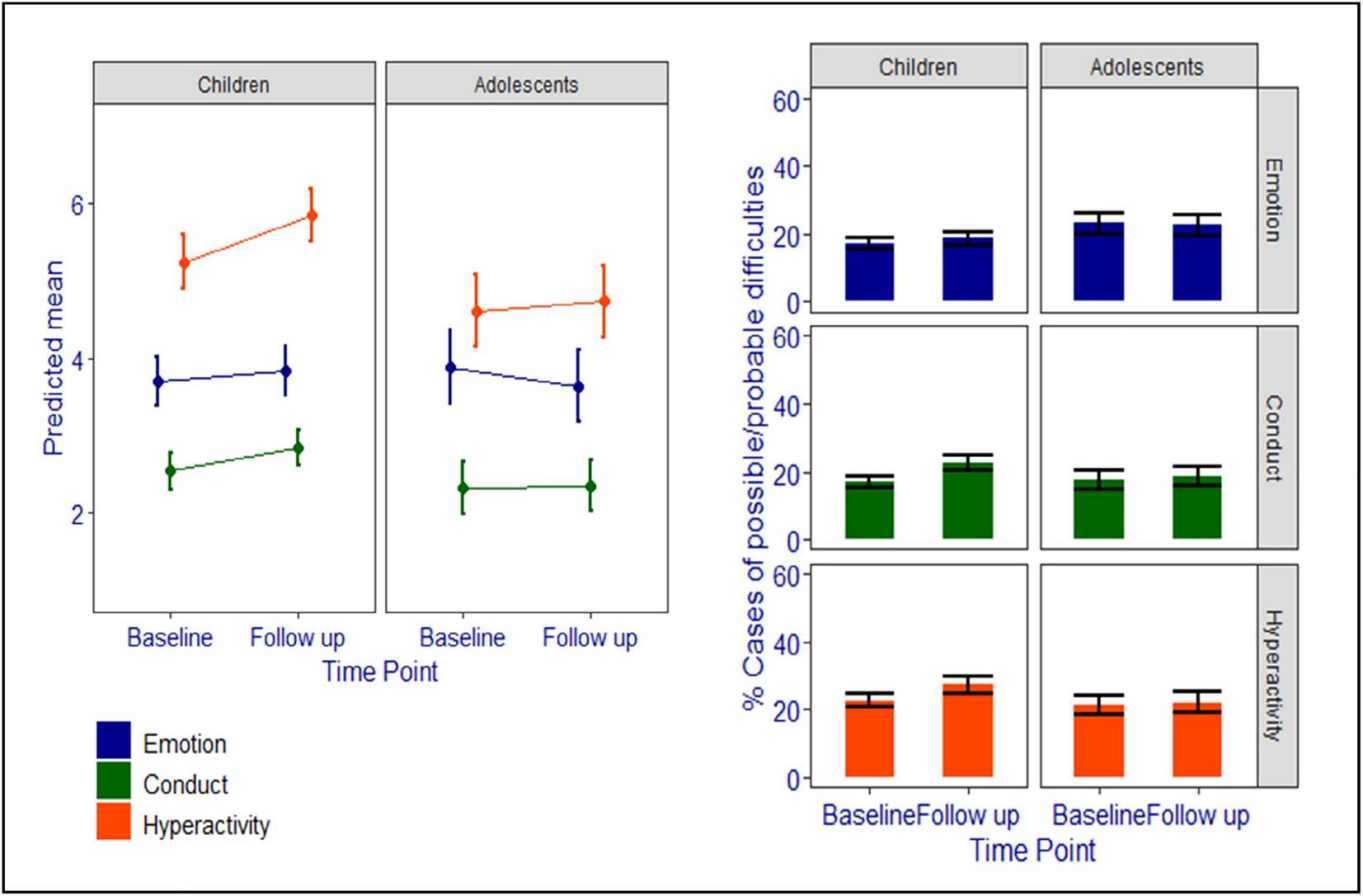
Estimated marginal means and % caseness for Strenghts and Difficulties Questionnaire emotional symptoms, conduct problems and hyperactivity/inattention from baseline to follow‐up, by age group

Between baseline and follow‐up, for children (age 4–10 years) there was a small increase in emotional symptoms and conduct problems and a larger increase in hyperactivity/inattention (standardised mean differences [SMD] of 0.05, 0.16 and 0.22, respectively). For adolescents (age 11–16 years), emotional symptoms reduced over time (SMD = −0.09), but there was little change in conduct problems (SMD = 0.02), and a small increase in hyperactivity/inattention (SMD = 0.04; see Figure 1).

Consistent with this pattern, for children, there were increases in caseness for emotion, conduct and hyperactivity/inattention (18.64%, 35.10% and 20.36% change, respectively); whereas the proportion of adolescents classified as a case did not change significantly for emotional, conduct or hyperactivity/inattention problems (2.89%, 7.71% and 4.20% change, respectively; see Figure 1).

Question 2. How did this vary on the basis of child gender, household income and family composition and presence of SENs/NDs?

**FIGURE 2:**
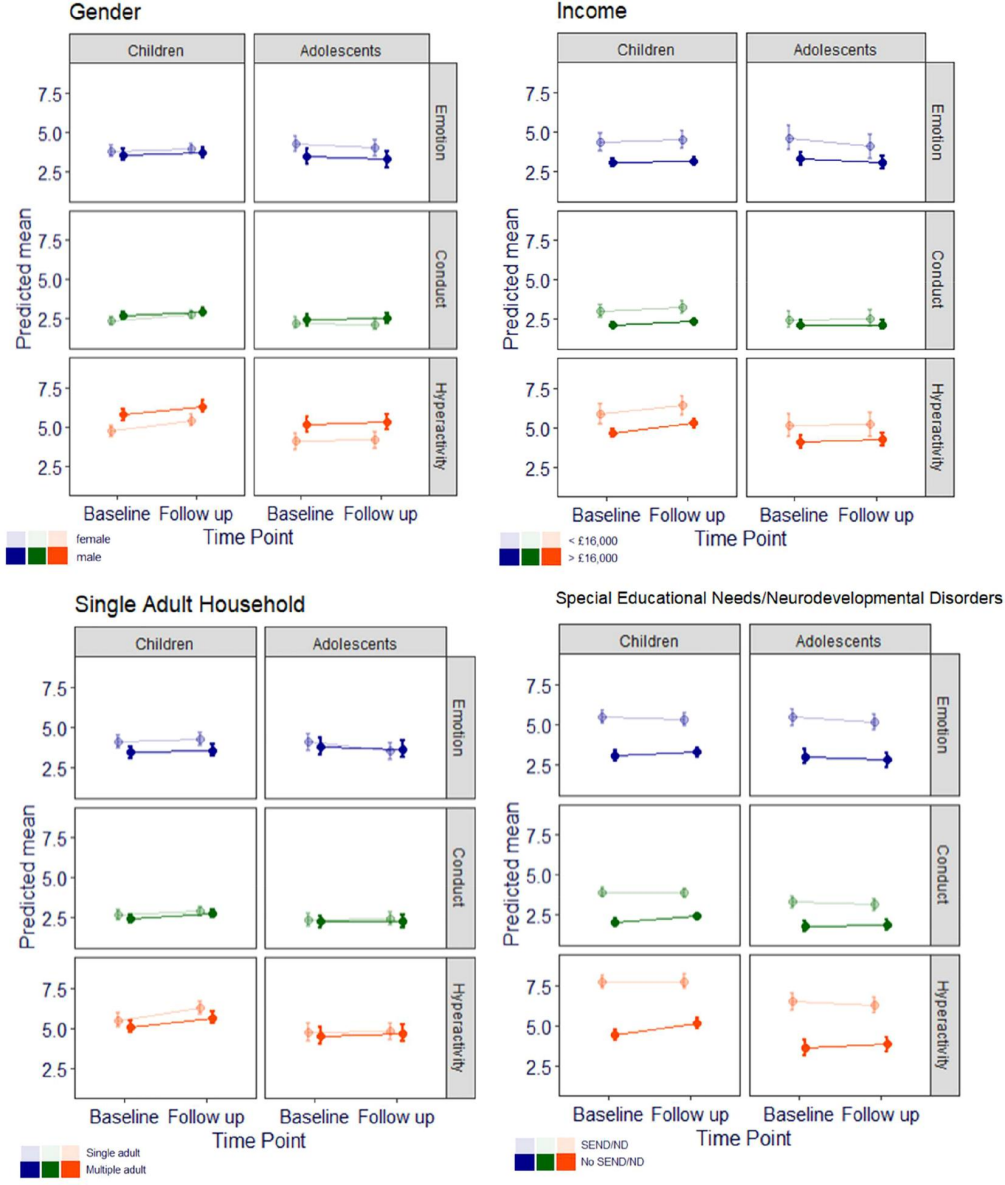
Predicted means for Strenghts and Difficulties Questionnaire emotional symptoms, conduct problems and hyperactivity/inattention from baseline to follow up, by age group and each moderator variable (with 95% CI error bars). CI, confidence interval

Table 2 presents the model results of the main effects of predictor variables and their interaction effects with time for parent/carer reported emotional symptoms, conduct problems and hyperactivity/inattention and for caseness. Figure 2 presents the change in time for each level of each predictor, split by age group. All means and confidence intervals for SDQ symptom scores, as well as percentages and percentage change for cases, can be found in Tables S1 and S2 (available as an online data supplement).

(i) Child gender

Across time points, compared to boys, girls had higher emotional symptoms and lower hyperactivity/inattention symptoms and caseness among both children and adolescents. In children, girls also had lower levels of conduct problems than boys. However, among the children, girls exhibited a greater increase in conduct scores over time than boys. For adolescents, there was a small increase among boys and a small reduction among girls. There were no significant interactions between time and gender for emotional symptoms or hyperactivity/inattention among children or adolescents. However, when caseness was considered, among children there was a significantly greater increase among girls than boys for possible/probable emotional, conduct and hyperactivity/inattention caseness. There were no significant interactions between gender and time for possible/probably caseness among adolescents.

(ii) Household income and family composition

Markedly elevated emotional and hyperactivity/inattention scores were found across time points for both children and adolescents in low income compared to high income families (and conduct for children), and this was also found for caseness for emotions and hyperactivity/inattention. However, changes in scores/caseness over time did not differ according to household income category.

Across time points, children living in single‐adult households had elevated emotional and hyperactivity/inattention symptoms but did not differ from those in multiple adult households on conduct symptoms or caseness on the emotional, conduct, and hyperactivity/inattention subscales. Adolescents did not differ significantly on any of the three subscales on the basis of single/multiple adult household. However, for adolescents (but not children) there was a significant interaction between adults in the household and time for emotional symptoms, with a greater reduction in emotion symptoms found among for adolescents from a single adult household compared to adolescents from a multiple adult household, and for hyperactivity/inattention caseness, reflecting a 9.1% increase in caseness among multiple adult households and a 11.55% decrease in single adult households. A similar pattern was found for conduct problems caseness for children, with a greater increase in caseness in multiple compared to single adult households. No other household status by time interactions were significant.

(iii) Child SENs/NDs

Both children and adolescents with SEN/ND had markedly elevated emotion, conduct, and hyperactivity/inattention scores and caseness compared to those without SEN/ND across time points. For children and adolescents there was a significant interaction between SEN/ND and time, reflecting a very small decrease for those with SEN/ND for SDQ conduct and hyperactivity/inattention and a small increase in scores/caseness for those without SEN/ND. For children, a similar pattern was also found for emotional symptoms and caseness, where those with SEN/ND experienced a significant decrease and those without SEN/ND experienced a significant increase.

## DISCUSSION

This study set out to explore how common mental health symptoms in children and adolescents changed (on the basis of parent/carer report) over a month of full lockdown in the United Kingdom in response to the COVID‐19 pandemic. The findings highlighted particular deteriorations in mental health symptoms among preadolescent children, which translated to a 10% increase in those meeting possible/probable caseness criteria for emotional symptoms, a 20% increase in hyperactivity/inattention, and a 35% increase in conduct problems. In contrast, changes among adolescents were smaller with a small reduction in emotional symptoms. Overall, there were few differences in change in symptoms or caseness over time according to demographic characteristics, with those at increased risk of mental health difficulties, such as those in low income households and those with SEN/ND, exhibiting elevated symptoms (and caseness) at both assessments. However, there were a few notable exceptions, in particular, among preadolescent children, there were greater increases in conduct symptoms and emotional, conduct and hyperactivity/inattention among girls than boys; whereas in adolescents, there were no differences in changes over time on the basis of gender. Two notable groups where scores decreased over time were adolescents from single‐parent households (in terms of emotional symptoms) and children and adolescents with SEN/ND (for conduct problems and hyperactivity/inattention).

Given the unprecedented context in which this study took place, it remains unclear why particular groups of children and young people experienced particular patterns of change in mental health symptoms and caseness. The finding that increases in mental health difficulties were most pronounced among primary school aged children may be surprising, given the known risk for the onset of mental health problems in adolescence (e.g., Kessler et al., 2005). However, on the other hand, increases in family stress caused by the demands of home‐schooling alongside working (NHS Digital, 2020) may have been a particular challenge for parents of younger children who would have been more reliant on parents for support with education, as well as generally monitoring, entertaining and providing for them throughout the day. On the other hand, adolescents may have been relatively independent during lockdown, and were also likely to have been able to better maintain peer relationships through, for example, online chats, messaging and gaming. The potential impact on both family stress and peer relationships on adjustment during lockdown will be a critical area for future research.

The increases in externalising (conduct, hyperactivity and inattention) problems across the age range are of particular concern, given the wide range of associated negative consequences for individuals, families and societies (Erskine et al., 2016). It will be important to carefully monitor this over time to understand to what extent they reflect particular challenges associated with the early lockdown period, and whether they resolve once children and young people are able to return to (some of) their normal activities or persist and require further support. Notably, however, emotional symptoms somewhat declined among adolescents. The lack of prepandemic data and day‐to‐day data right from the start of the pandemic makes this difficult to interpret, as it is possible that, for example, adolescents' levels of emotional symptoms had increased prior to the start of this study and we saw a gradual return to ‘normal’ levels. Alternatively, it is possible that aspects of lockdown brought some benefits to participating adolescents, particularly due to a reduction in academic or social pressures (which are both known to be high among adolescents; e.g., Peña‐López, 2016). Whilst our findings are based on parent/carer report, at least one other study has reported a reduction in adolescent self‐reported anxiety levels among year 9 (13–14 year olds) from pre to during pandemic assessments (Widnall et al., 2020). Notably we also saw particular reductions in emotional symptoms among adolescents from single‐adult households and externalising problems among children and adolescents with SEN/ND. It is important to recognise that these groups had elevated mental health symptoms throughout, however it appears that, at least for some children and young people lockdown may have eased some challenging areas of life. These findings are consistent with others that have emerged during the pandemic that have highlighted particular groups of young people who have reported that their mental health benefited during lockdown (Mansfield et al., 2020; Mind, 2020), for example, due to enjoying more time with family members (Levita, 2020) and having more opportunities to engage in valued activities (The Children's Society, 2020).

While we do not have prepandemic data so cannot comment on, for example, changes in the prevalence of mental health problems because of the pandemic, these findings do give an indication of how mental health changed for children and young people within the first pandemic‐related lockdown in the United Kingdom. This has implications both for understanding the potential impact of such measures and for interpreting findings from other studies that have compared outcomes in prelockdown assessments to those collected at a particular point in time postlockdown. It is also important to highlight that, in order to be able use comparable measures across the 4–16 years age range, we relied on parent or carer reported mental health symptoms. Predicting caseness using the SDQ is improved with teacher as well as parent report (Goodman et al., 2000); however, this was not feasible while schools were closed during the pandemic. We are reassured by consistent patterns of findings with other studies that have examined adolescent self‐reported mental health symptoms (Widnall et al., 2020), however we do need to acknowledge the possibility that parents/carers may not have been aware of the full extent of any, particularly emotional, symptoms (e.g., Salbach‐Andrae et al., 2009). Indeed, the elevation in externalising problems (e.g., arguments) seen across the age range may reflect broader distress observed in the form of behavioural disturbance (Angold & Costello, 1993). It is also important to highlight that at the first assessment, the SDQ requires that symptoms be rated over the past 6 months, which then changes to the past month at subsequent follow‐up time periods. Thus, although parents' ratings at follow‐up are of their child's symptoms during the lockdown period, the baseline ratings cover a large time span, which should be taken into account when interpreting changes over time. Furthermore, while we examined commonly occurring mental health symptoms for this age range, using a well‐validated screening instrument, we did not assess the presence of mental health disorders against the diagnostic criteria of international standard classifications such as ICD‐10 (World Health Organization, 1992). Further research is also required to understand other mental health difficulties, such as those related to sleep or eating difficulties.

It is also important to highlight that the study population was not a representative sample, and there was clear bias towards more affluent families from White British backgrounds. Given the markedly elevated levels of mental health symptoms and caseness found among children and young people in low income households within our study, we expect that the levels of difficulties we have reported here are likely an under‐estimation of the extent of difficulties experienced more broadly in the community, and detection of predictors of change in mental health symptoms over time may have been limited by relatively small samples among some groups. Indeed, the very small samples within, for example, individual ethnic groups unfortunately meant that we were limited to combining children and adolescents from Black, Asian and ethnic minority backgrounds in to one category, which is a clear limitation given the very different experiences during the pandemic (Levita, 2020). Other factors such as the children and families' experience of COVID‐19, parental employment status (including whether they were a key worker, working out of the home and in relatively high risk environments) and child school attendance will also be important to consider in future investigations.

This rapid longitudinal study in response to the first COVID‐19 lockdown in the United Kingdom has highlighted deterioration in parent or carer reported externalising behaviours among participating children and, to a lesser extent, adolescents over 1 month of lockdown. While emotional symptoms also increased among preadolescents in this study, there was a small decrease among adolescents, and this was also the case for externalising problems among children and adolescents with SENs. As such the findings highlight important areas of concern in terms of the potential impact of the first national lockdown on children and young people's adjustment. It will be important to further track the trajectories of mental health of children and young people over the course of the pandemic beyond early lockdown, as schools reopen, as further regional and national lockdowns occur and the economic impacts are more keenly felt. Developing an understanding of who has been most severely affected by the pandemic, and in what ways, is crucial to target effective support where it is most needed.

## CONFLICT OF INTERESTS

The authors declare that there are no conflict of interests.

## AUTHOR CONTRIBUTIONS

Polly Waite and Cathy Creswell: *conceptualisation, funding acquisition, investigation, methodology, project administration, supervision, writing‐original draft, writing‐review & editing*. Samantha Pearcey: *data curation, formal analysis, project administration, writing‐original draft, writing‐review & editing*. Adrienne Shum and Jasmine Raw: *data curation, project administration, writing‐original draft, writing‐review & editing*. Praveetha Patalay: *conceptualisation, funding acquisition, methodology, supervision, writing‐review & editing*.

## Supporting information

Table S1

## Data Availability

The research materials can be accessed by contacting the corresponding author. At the end of the study the datasets will be made open access.
