# Supplementary material for: How did the mental health symptoms of children and adolescents change over early lockdown during the COVID‐19 pandemic in the UK?
Source: JCPP Adv. 2021 Apr 28;1(1):e12009. doi: 10.1111/jcv2.12009 (PMC8206715; doi:10.1111/jcv2.12009)
Supplement: Supplementary file 1 — Table S1 [file JCV2-1-e12009-s001.docx]

**Supporting information - Examining changes in parent-reported child and adolescent mental health throughout the UK’s first COVID-19 national lockdown – by Waite *et al.***

**Table S1.**

*Estimated marginal means and confidence intervals for parent/carer reported emotional, conduct and hyperactivity/inattention symptoms at baseline and follow up.*

|  |  | **SDQ Emotion** | | **SDQ Conduct** | | **SDQ Hyperactivity/inattention** | |
| --- | --- | --- | --- | --- | --- | --- | --- |
|  |  | *Baseline* | *Follow up* | *Baseline* | *Follow up* | *Baseline* | *Follow up* |
| *Children* | | | | | | | |
| Overall | | 3.70 [3.38, 4.02] | 3.84 [3.52, 4.16] | 2.54 [2.31, 2.77] | 2.83 [2.60, 3.06] | 5.25 [4.91, 5.59] | 5.86 [5.51, 6.20] |
| Gender | Males | 3.58 [3.24, 3.91] | 3.72 [3.38, 4.06] | 2.69 [2.44, 2.93] | 2.92 [2.68, 3.16] | 5.77 [5.41, 6.13] | 6.30 [5.94, 6.67] |
|  | Females | 3.83 [3.49, 4.17] | 3.95 [3.61, 4.30] | 2.38 [2.13, 2.63] | 2.75 [2.50, 2.99] | 4.73 [4.36, 5.10] | 5.14 [5.04, 5.78] |
| Income | < £16,000 | 4.33 [3.75, 4.90] | 4.54 [3.97, 5.11] | 2.99 [2.58, 3.40] | 3.24 [2.83, 3.66] | 5.86 [5.25, 6.48] | 6.41 [5.80, 7.02] |
|  | > £16,000 | 3.04 [2.80, 3.28] | 3.17 [2.94, 3.41] | 2.10 [1.93, 2.27] | 2.40 [2.23, 2.57] | 4.66 [4.41, 4.92] | 5.27 [5.02, 5.53] |
| Single Adult | Single adult | 4.13 [3.72, 4.53] | 4.27 [3.87, 4.68] | 2.70 [2.40, 2.99] | 2.90 [2.61, 3.20] | 5.48 [5.05, 5.92] | 6.27 [5.83, 6.71] |
|  | Multiple adult | 3.45 [3.10, 3.79] | 3.58 [3.24, 3.93] | 2.46 [2.21, 2.71] | 2.77 [2.52, 3.02] | 5.07 [4.70, 5.44] | 5.65 [5.28, 6.02] |
| SEN/ND | SEN/ND | 5.46 [5.06, 5.85] | 5.33 [4.93, 5.72] | 3.92 [3.64, 4.20] | 3.90 [3.62, 4.18] | 7.70 [7.29, 8.11] | 7.76 [7.35, 8.17] |
|  | No SEN/ND | 3.10 [2.78, 3.41] | 3.27 [2.96, 3.59] | 2.07 [1.84, 2.29] | 2.41 [2.19, 2.64] | 4.42 [4.09, 4.75] | 5.11 [4.78, 5.44] |
| *Adolescents* | | | | | | | |
| Overall | | 3.89[3.42, 4.35] | 3.64[3.17, 4.11] | 2.32 [1.99, 2.65] | 2.34 [2.01, 2.68] | 4.61 [4.14, 5.08] | 4.73 [4.26, 5.20] |
| Gender | Males | 3.49 [2.99, 3.98] | 3.28 [2.78, 3.77] | 2.42 [2.07, 2.77] | 2.52 [2.17, 2.87] | 5.17 [4.67, 5.66] | 5.33 [4.83, 5.82] |
|  | Females | 4.29 [3.78, 4.79] | 4.01 [3.50, 4.51] | 2.22 [1.87, 2.58] | 2.16 [1.81, 2.52] | 4.06 [3.55, 4.56] | 4.14 [3.63, 4.65] |
| Income | < £16,000 | 4.64 [3.89, 5.39] | 4.08 [3.33, 4.83] | 2.48 [1.95, 3.00] | 2.56 [2.04, 3.09] | 5.12 [4.38, 5.87] | 5.19 [4.45, 5.94] |
|  | > £16,000 | 3.28 [2.87, 3.68] | 3.06 [2.65, 3.46] | 2.13 [1.85, 3.42] | 2.16 [1.87, 2.44] | 4.12 [3.72, 4.53] | 4.25 [3.85, 4.65] |
| Single Adult | Single adult | 4.08 [3.35, 4.63] | 3.51 [2.96, 4.06] | 2.35 [1.96, 2.74] | 2.44 [2.05, 2.82] | 4.74 [4.19, 5.29] | 4.78 [4.23, 5.33] |
|  | Multiple adult | 3.83 [3.31, 4.35] | 3.65 [3.13, 4.17] | 2.27 [1.91, 2.64] | 2.29 [1.92, 2.65] | 4.52 [4.00, 5.04] | 4.66 [4.14, 5.19] |
| SEN/ND | SEN/ND | 5.47 [4.96, 5.98] | 5.15 [4.64, 5.65] | 3.32 [2.96, 3.96] | 3.14 [2.77, 3.50] | 6.48 [5.98, 6.98] | 6.26 [5.76, 6.75] |
|  | No SEN/ND | 3.01 [2.55, 3.46] | 2.79 [2.33, 3.24] | 1.78 [1.46, 2.11] | 1.87 [1.54, 2.20] | 3.61 [3.16, 4.06] | 3.83 [3.38, 4.28] |

Statistical significance for the change over time for each moderator is presented as an interaction in Table 2 within the manuscript.

**Table S2.**

Percentages and confidence intervals of participants who are a ‘possible’/‘probable’ case with parent/carer reported emotional, conduct and hyperactivity/inattention symptoms at baseline and follow-up.

|  |  | **SDQ Emotion** | | | | | **SDQ Conduct** | | | | | **SDQ Hyperactivity/inattention** | | | | |
| --- | --- | --- | --- | --- | --- | --- | --- | --- | --- | --- | --- | --- | --- | --- | --- | --- |
|  |  | *Baseline* | | *Follow up* | | *% change* | *Baseline* | | *Follow up* | | *% change* | *Baseline* | | *Follow up* | | *% change* |
|  |  | *Cases*  *(%)* | *95% CI* | *Cases*  *(%)* | *95% CI* |  | *Cases*  *(%)* | *95% CI* | *Cases*  *(%)* | *95% CI* |  | *Cases*  *(%)* | *95% CI* | *Cases*  *(%)* | *95% CI* |  |
| *Children* | | | | | | | | | | | | | | | |  |
| Overall | | 17.00 | 15.14, 18.87 | 18.64 | 16.68, 20.59 | 9.65% | 16.84 | 14.99, 18.69 | 22.75 | 20.58, 24.92 | 35.10% | 22.69 | 20.53, 24.86 | 27.31 | 24.92, 29.69 | 20.36% |
| Gender | Males | 17.90 | 15.24, 20.57 | 17.69 | 15.04, 20.33 | -1.17% | 19.87 | 17.06, 22.68 | 24.67 | 21.52, 27.82 | 24.16% | 29.26 | 25.82, 32.70 | 31.55 | 27.97, 35.13 | 7.83% |
|  | Females | 15.83 | 13.24, 18.41 | 19.46 | 16.58, 22.34 | 22.93% | 13.60 | 11.22, 15.98 | 20.75 | 17.77, 23.73 | 52.57% | 15.59 | 13.03, 18.16 | 22.51 | 19.40, 25.62 | 44.39% |
| Income | < £16,000 | 40.51 | 26.65, 54.37 | 44.30 | 29.79, 58.82 | 9.36% | 31.65 | 19.44, 43.85 | 44.30 | 29.79, 58.82 | 39.97% | 48.10 | 32.97, 63.24 | 51.90 | 36.17, 67.63 | 7.90% |
|  | > £16,000 | 15.91 | 14.07, 17.75 | 17.44 | 15.51, 19.37 | 9.62% | 16.15 | 14.29, 18.00 | 21.74 | 19.58, 23.91 | 34.61% | 21.51 | 19.35, 23.66 | 26.16 | 23.78, 28.55 | 21.62% |
| Single Adult | Single adult | 27.18 | 20.20, 34.17 | 28.16 | 21.04, 35.27 | 3.61% | 26.21 | 19.36, 33.07 | 27.67 | 20.62, 34.72 | 5.57% | 29.13 | 21.88, 36.37 | 37.38 | 29.14, 45.62 | 28.32% |
|  | Multiple adult | 15.67 | 13.77, 17.56 | 17.39 | 15.39, 19.39 | 10.98% | 15.61 | 13.72, 17.50 | 22.10 | 19.83, 24.37 | 41.58% | 21.85 | 19.59, 24.11 | 25.99 | 23.52, 28.46 | 18.95% |
| SEN/ND | SEN/ND | 55.56 | 46.27, 64.84 | 49.79 | 41.01, 58.58 | -10.39% | 51.03 | 42.14, 59.92 | 48.15 | 39.52, 56.78 | -5.64% | 78.60 | 67.53, 89.68 | 75.31 | 64.47, 86.15 | -4.19% |
|  | No SEN/ND | 10.89 | 9.32, 12.47 | 13.70 | 11.92, 15.48 | 25.80% | 11.42 | 9.80, 13.03 | 18.72 | 16.61, 20.83 | 63.92% | 13.83 | 12.04, 15.62 | 19.70 | 17.54, 21.87 | 42.44% |
| *Adolescents* | | | | | | | | | | | | | | | |  |
| Overall | | 23.19 | 20.11, 26.27 | 22.52 | 19.48, 25.56 | -2.89% | 17.39 | 14.74, 20.04 | 18.73 | 15.97, 21.49 | 7.71% | 21.18 | 18.24, 24.12 | 22.07 | 19.07, 25.08 | 4.20% |
| Gender | Males | 19.74 | 15.81, 23.67 | 18.46 | 14.66, 22.25 | -6.74% | 18.88 | 15.04, 22.72 | 21.67 | 17.55, 25.80 | 14.78% | 25.97 | 21.43, 30.50 | 27.04 | 22.41, 31.67 | 4.12% |
|  | Females | 26.56 | 21.71, 31.40 | 26.08 | 21.28, 30.88 | -1.81% | 15.79 | 12.10, 19.48 | 15.79 | 12.10, 19.48 | 0.00% | 16.03 | 12.31, 19.75 | 16.75 | 12.94, 20.55 | 4.49% |
| Income | < £16,000 | 50.82 | 33.11, 68.53 | 42.62 | 26.43, 58.81 | -16.14% | 24.59 | 12.40, 36.78 | 24.59 | 12.40, 36.78 | 0.00% | 42.62 | 26.43, 58.81 | 37.71 | 22.50, 52.91 | -11.52% |
|  | > £16,000 | 21.17 | 18.13, 24.22 | 21.05 | 18.02, 24.09 | -0.57% | 16.87 | 14.17, 19.57 | 18.30 | 15.48, 21.12 | 8.48% | 19.62 | 16.69, 22.54 | 20.93 | 17.91, 23.96 | 6.68% |
| Single Adult | Single adult | 27.74 | 19.60, 35.88 | 26.45 | 18.51, 34.39 | -4.65% | 20.00 | 13.14, 26.86 | 19.36 | 12.61, 26.10 | -3.20% | 27.74 | 19.60, 35.88 | 24.52 | 16.88, 32.15 | -11.61% |
|  | Multiple adult | 22.24 | 18.92, 25.55 | 21.70 | 18.43, 24.97 | -2.43% | 16.85 | 13.98, 19.71 | 18.60 | 15.58, 21.62 | 10.39% | 19.81 | 16.69, 22.93 | 21.56 | 18.30, 24.83 | 8.83% |
| SEN/ND | SEN/ND | 55.77 | 45.71, 65.83 | 51.92 | 42.23, 61.62 | -6.90% | 40.39 | 31.86, 48.91 | 35.58 | 27.59, 43.57 | -11.91% | 59.62 | 49.21, 70.02 | 55.29 | 45.28, 65.30 | -7.26% |
|  | No SEN/ND | 13.35 | 10.73, 15.98 | 13.64 | 10.99, 16.30 | 2.17% | 10.45 | 8.16, 12.75 | 13.64 | 10.99, 16.30 | 30.53% | 9.58 | 7.39, 11.77 | 12.05 | 9.57, 14.53 | 25.78% |

Statistical significance for the change over time for each moderator is presented as an interaction in Table 2 within the manuscript.
